# Anesthetic Considerations in Pediatric Corneal Neurotization: A Case Report

**DOI:** 10.7759/cureus.93566

**Published:** 2025-09-30

**Authors:** José Enrique Díaz Vázquez, Mauricio Muleiro-Alvarez, Alejandra Botero-Benítez, María Alejandra Juárez Tirado, Javier Burgos Cárdenas, Neyra Xiomara Pérez Garcés, Tania Albavera-Giles, Alexandro Aguilera, Nicolás Kahuam-López, Arturo Ramirez-Miranda, Guillermo Raul Vera-Duarte

**Affiliations:** 1 Anesthesiology, Instituto de Oftalmología Fundación Conde de Valenciana es una Institución de Asistencia Privada, Mexico City, MEX; 2 Cornea and Refractive Surgery, Instituto de Oftalmología Fundación Conde de Valenciana es una Institución de Asistencia Privada, Mexico City, MEX; 3 Plastic Surgery, Medpoint Plastic Surgery Clinic, Polanco, Mexico City, MEX

**Keywords:** intraoperative management, multimodal analgesia, neurotization, pediatric anesthesia, postoperative care, preoperative anxiety reduction, preoperative care

## Abstract

Congenital corneal anesthesia (CCA) is a rare pediatric disorder characterized by absent or markedly reduced corneal sensation, predisposing to neurotrophic keratopathy and vision-threatening complications. Corneal neurotization has emerged as a surgical option to restore corneal innervation, but anesthetic considerations in pediatric cases remain scarcely reported. We present the anesthetic management of a six-year-old female child with CCA and recurrent herpetic keratitis following deep anterior lamellar keratoplasty, who underwent indirect corneal neurotization with a sural nerve graft. Anesthetic care focused on preoperative anxiety reduction, airway safety, multimodal analgesia, and limited neuromuscular blockade to facilitate intraoperative neuromonitoring. Standard monitoring included electrocardiography, non-invasive blood pressure, pulse oximetry, capnography, temperature, and ulnar nerve twitch assessment. Perioperative antiviral prophylaxis (oral acyclovir) was maintained, and ondansetron was administered for PONV prophylaxis. The four-hour procedure was uneventful, with stable hemodynamics and adequate postoperative pain control. Pain was assessed using the FLACC (Face, Legs, Activity, Cry, Consolability) scale, which remained at a level of <3/10 throughout the first 24 hours. This report highlights the importance of tailored anesthetic strategies in pediatric neurotization procedures, balancing surgical requirements with age-specific physiological considerations.

## Introduction

Congenital corneal anesthesia (CCA) is a rare [1], typically bilateral condition characterized by markedly reduced or absent corneal sensation, which predisposes young patients to neurotrophic keratopathy (NK), which can manifest as dry geographic spots on the corneal surface, evident stromal lysis, and recurrent epithelial defects that lead to recurrent infections [2]. It may occur without an identifiable cause or in association with neurological disorders [3]. If NK is not promptly diagnosed and treated, it can progress to corneal ulceration and even perforation [4]. 

Conventional management is directed at complication prevention and includes preservative-free lubricants, autologous serum eye drops, and, in refractory cases, surgical interventions such as tarsorrhaphy, amniotic membrane transplantation, or keratoplasty [5]. Nevertheless, these strategies do not address the underlying loss of corneal innervation. Since its description by Terzis et al. [6], corneal neurotization has emerged as a promising surgical option to restore corneal sensation and ocular surface integrity. Techniques are classified as direct or indirect. The first technique is performed by transferring a nerve from its original position to innervate the cornea (direct corneal neurotization), and on the other hand, it can also be done by using a nerve graft and connecting it to the cornea (indirect corneal neurotization) [7,8]. Whichever technique is chosen, both involve a relatively long surgical procedure; therefore, it is a must to use an adequate anesthetic technique that allows the evaluation of all parameters during surgery.

Despite the growing number of reports on surgical outcomes, anesthetic considerations in pediatric neurotization remain scarcely described. Particular challenges include age-specific physiology, airway safety, perioperative analgesia, and the prolonged duration of surgery. In addition, neurotrophic keratopathy represents a progressive degenerative disease caused by impaired corneal innervation, leading to persistent epithelial defects, ulceration, scarring, and vision loss; these pathophysiologic features inform perioperative protection of the ocular surface. Herein, we report the anesthetic management of a child with CCA undergoing indirect corneal neurotization with a sural nerve graft, highlighting strategies implemented to optimize perioperative safety and surgical success.

## Case presentation

A six-year-old female child weighing 20 kg, with a diagnosis of CCA, presented with a history of recurrent episodes of herpetic keratitis in the left eye. She had previously undergone deep anterior lamellar keratoplasty (DALK) for a corneal leucoma; however, multiple recurrences of herpetic keratitis occurred postoperatively despite adequate management, which included prophylactic oral acyclovir, preservative-free artificial tears, and regular ocular surface protection strategies. The last recurrence occurred three months before surgery. Perioperative antiviral prophylaxis with oral acyclovir (800 mg five times daily) was maintained preoperatively and continued postoperatively to reduce the risk of herpes simplex virus (HSV) reactivation. Persistent corneal hypoesthesia and progressive ocular surface compromise led to the indication of indirect corneal neurotization with a sural nerve graft.

Preoperative evaluation revealed a Mallampati Class I airway with normal cervical spine mobility, adequate mouth opening, and normal dentition. Immediate preoperative vital signs were heart rate 92 beats per minute (bpm), blood pressure 100/60 mmHg, respiratory rate 20 breaths/minute, and oxygen saturation 99% on room air. Routine laboratory tests, including complete blood count and coagulation profile, were within normal limits. Fasting status was confirmed (six hours for solids and two hours for clear fluids). To reduce preoperative anxiety, oral midazolam (0.5 mg/kg) was administered 30 minutes before surgery, and parental presence was permitted during induction. In the operating room, standard monitoring was applied. General anesthesia was induced with sevoflurane (8% in oxygen) via face mask, which allowed atraumatic IV cannulation; this was followed by intravenous propofol (2 mg/kg) to deepen anesthesia, stabilize hemodynamics during laryngoscopy, and reduce the risk of emergence agitation, and fentanyl (2 mcg/kg). Rocuronium (0.6 mg/kg) was administered to facilitate endotracheal intubation with a 5.0 mm cuffed tube. Anesthesia was maintained with sevoflurane (1.5-2%) in a 50:50 oxygen-air mixture, with additional fentanyl boluses (1 mcg/kg) as required, guided by clinical signs of inadequate analgesia (increase >20% from baseline in heart rate or blood pressure). Because intraoperative neuromonitoring was necessary, neuromuscular blockade was minimized beyond induction and monitored using an ulnar nerve twitch stimulator; 4/4 twitches were present during functional nerve assessment to ensure reliable evaluation of donor-graft coaptation.

**Figure 1:**
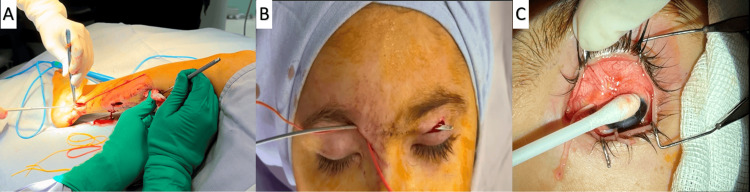
Surgical steps of indirect corneal neurotization with a sural nerve graft. A: Harvesting of the sural nerve graft from the lower limb; B: Creation of a subcutaneous tunnel in the periocular region and passage of the nerve graft; C: Separation and implantation of nerve fascicles into the perilimbal corneal stroma. Written informed consent was obtained from the patient’s legal guardian for use of both identifying (e.g., face photographs) and/or non-identifying (e.g., diagnostic images) images in open-access publication.

The surgical procedure consisted of harvesting the sural nerve from the lower limb through a longitudinal incision, providing a graft of sufficient length (approximately 12 cm). Two surgical teams (ophthalmology and plastic surgery) worked in parallel to reduce operative time. The graft, harvested from the leg, was not tunneled from the leg to the face; instead, it was prepared and then subcutaneously tunneled within the periorbital region, from the supraorbital/supratrochlear area across the nasal bridge, where fascicles were separated and implanted circumferentially into perilimbal corneoscleral pockets (Figure 1). Lactated Ringer’s solution was infused at 4 mL/kg/hour, adjusted for surgical losses. Estimated blood loss was <20 mL, urine output was negligible, and the total surgical time was approximately four hours, with stable hemodynamics maintained throughout (heart rate 80-100 bpm; blood pressure 90/50-110/60 mmHg). Qualitative sensory evaluation of the sural harvest site was performed postoperatively using light touch and blunt stimulus to confirm protective sensation.

**Figure 2:**
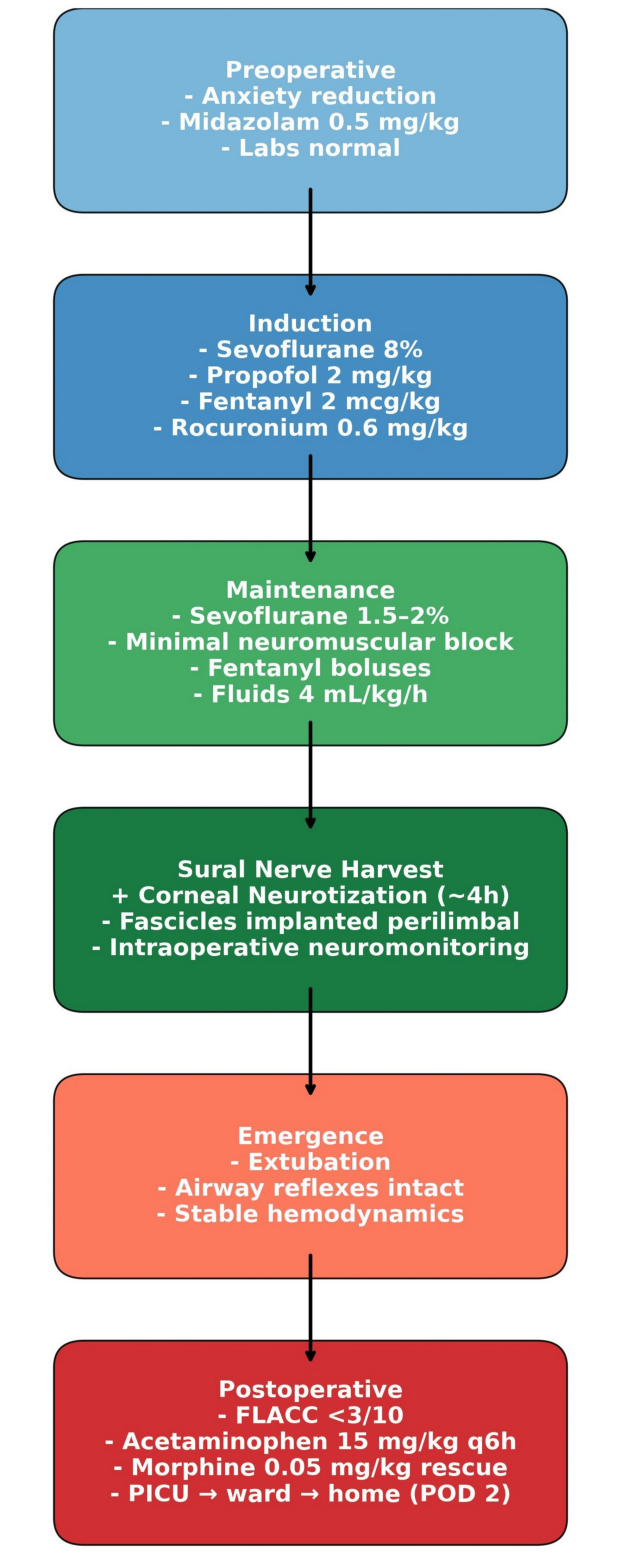
Anesthetic timeline in pediatric corneal neurotization with sural nerve graft. Flowchart summarizing preoperative anxiolysis, inhalational induction with sevoflurane, and IV transition with propofol, neuromuscular management with ulnar nerve twitch monitoring, intraoperative analgesia (fentanyl triggers), fluid therapy, PONV prophylaxis (ondansetron), and postoperative ICU monitoring with continuous SpO₂. PONV: postoperative nausea and vomiting; SpO₂: oxygen saturation; FLACC: Face, Legs, Activity, Cry, Consolability; PICU: pediatric intensive care unit; POD: postoperative day

At the end of surgery, extubation was performed in the operating room once adequate spontaneous ventilation and airway reflexes were confirmed. Neuromuscular recovery was complete, and no reversal agent was required. Postoperative analgesia included intravenous acetaminophen (15 mg/kg every six hours) and morphine (0.05 mg/kg as rescue). Ondansetron (0.1 mg/kg IV) was administered intraoperatively for postoperative nausea and vomiting prophylaxis; no emetic episodes occurred. Pain was assessed using the FLACC scale (Face, Legs, Activity, Cry, Consolability) [9], remaining consistently <3/10 during the first 24 hours. The patient was admitted to the pediatric intensive care unit for 24 hours with continuous pulse oximetry due to prolonged anesthesia and opioid exposure, transferred to the general ward on postoperative day 1, and discharged home on day 2 with stable ocular and systemic conditions (Figure 2).

## Discussion

This case demonstrates successful anesthetic management of a pediatric patient undergoing indirect corneal neurotization and underscores procedure-specific challenges beyond general pediatric anesthesia. The indications for this procedure are typically the presence of nerve damage and as a treatment of neuropathy associated with HSV and congenital corneal anesthesia, and the primary aim for taking this decision is to restore corneal sensitivity to prevent ulcers and perforations. In this context, we specified the monitored parameters, ECG, blood pressure, oxygen saturation (SpO₂), capnography, temperature, and ulnar nerve twitch, rather than using broad terms. General anesthesia is almost always required for the procedure. Advantages of performing this surgical procedure, as demonstrated by previous studies, are recovery of corneal sensitivity and objective epithelial improvement in most patients and toddlers; results may be better due to their greater capacity for nerve regeneration [10]. This case challenges the prolonged microsurgical duration, minimizing neuromuscular blockade to permit functional nerve assessment, maintaining stable hemodynamics during delicate coaptation, and implementing strict postoperative nausea and vomiting (PONV) prophylaxis to avoid suture disruption.

Anesthetic management in this setting must account for specific pediatric considerations, including airway safety, intraoperative neuromonitoring, and postoperative pain control. The anesthetic agents selected in this case were based on established safety profiles and advantages reported in comparative studies. Propofol was used for induction, given its association with a reduced incidence of emergence agitation, postoperative nausea, and pain in young children [11]. Sevoflurane was selected for maintenance because of its rapid onset and offset, facilitating stable intraoperative conditions and smoother recovery [11]. 

Neuromuscular blockade was achieved with rocuronium to ensure optimal intubating conditions while minimizing subsequent doses to preserve the accuracy of intraoperative nerve monitoring. Evidence supports the use of rocuronium in procedures requiring functional testing, as it does not interfere with evoked potentials when appropriately titrated [12,13]. Furthermore, randomized pediatric trials have shown that rocuronium provides intubating conditions comparable or superior to succinylcholine, with the added advantage of greater hemodynamic stability and fewer adverse effects [14]. Conversely, inadequate dosing may result in incomplete paralysis, spontaneous motor activity, and compromised neurophysiological recordings. To maintain reliable functional nerve testing, neuromuscular blockade was minimized after induction and titrated using an ulnar nerve twitch monitor; 4/4 twitches were documented during donor-graft assessment, and sugammadex was not required as recovery was spontaneous by the end of surgery. Given the risk that emesis may disrupt delicate corneal sutures, ondansetron was administered prophylactically, and no PONV was observed. The decision for 24-hour ICU observation with continuous SpO₂ monitoring was based on the duration of anesthesia and opioid exposure, aiming to reduce the risk of unrecognized respiratory depression in the immediate postoperative period. Preoperative anxiolysis, combined with parental presence, facilitated a smooth induction in accordance with pediatric anesthesia practices. Although dexmedetomidine may be considered in similar cases for its anxiolytic and analgesic properties without reported neurodevelopmental toxicity [15], it was not required in this patient.

Preoperative administration of midazolam and parental presence effectively reduced anxiety, facilitating a smoother induction. Postoperative analgesia was achieved through a multimodal regimen, minimizing opioid use and thereby reducing the risk of respiratory complications. The need for ongoing research into optimal anesthetic strategies for pediatric neurosurgery is underscored by concerns regarding potential neurodevelopmental effects of prolonged general anesthesia before the age of seven. Reported risks include cognitive and behavioral impairments, developmental delays, learning difficulties, autism spectrum features, and, in some cases, associations with cerebral palsy [16]. Although the precise mechanisms and long-term outcomes remain under investigation, these concerns highlight the necessity for judicious anesthetic use in young children.

## Conclusions

This case highlights the value of a tailored anesthetic approach in pediatric corneal neurotization. Comprehensive preoperative planning, careful intraoperative monitoring, and strategic multimodal analgesia were key to achieving a favorable outcome. Specific measures included perioperative antiviral prophylaxis, limited neuromuscular blockade with ulnar nerve twitch guidance to permit functional testing, PONV prophylaxis, and 24-hour ICU observation with continuous SpO₂ monitoring. Further research is warranted to refine anesthetic protocols and establish evidence-based guidelines for pediatric neurotization procedures.

## References

[1] Frarchi M, Bouslous N, Laakri J, Ajbar K, Moustaine O (2025). Congenital corneal anesthesia secondary to brainstem ischemia as a rare complication of neonatal hypoxic-ischemic encephalopathy. Cureus.

[2] Jayarajan AP, Sharma A, Sharma R, Nirankari VS, Narayana S, Christy JS (2022). Congenital corneal anesthesia: a case series. Indian J Ophthalmol.

[3] Gelzinis A, Simonaviciute D, Krucaite A, Buzzonetti L, Dollfus H, Zemaitiene R (2022). Neurotrophic keratitis due to congenital corneal anesthesia with deafness, hypotonia, intellectual disability, face abnormality and metabolic disorder: a new syndrome?. Medicina (Kaunas).

[4] Mantelli F, Nardella C, Tiberi E, Sacchetti M, Bruscolini A, Lambiase A (2015). Congenital corneal anesthesia and neurotrophic keratitis: diagnosis and management. Biomed Res Int.

[5] Versura P, Giannaccare G, Pellegrini M, Sebastiani S, Campos EC (2018). Neurotrophic keratitis: current challenges and future prospects. Eye Brain.

[6] Terzis JK, Dryer MM, Bodner BI (2009). Corneal neurotization: a novel solution to neurotrophic keratopathy. Plast Reconstr Surg.

[7] Dragnea DC, Krolo I, Koppen C, Faris C, Van den Bogerd B, Ní Dhubhghaill S (2023). Corneal neurotization-indications, surgical techniques and outcomes. J Clin Med.

[8] Samoilă O, Samoilă L, Petrescu L (2025). Corneal neurotization, recent progress, and future perspectives. Biomedicines.

[9] Merkel SI, Voepel-Lewis T, Shayevitz JR, Malviya S (1997). The FLACC: a behavioral scale for scoring postoperative pain in young children. Pediatric nursing.

[10] Saini M, Jain A, Vanathi M, Kalia A, Saini K, Gupta P, Gaur N (2024). Current perspectives and concerns in corneal neurotization. Indian J Ophthalmol.

[11] Zhao Y, Qin F, Liu Y, Dai Y, Cen X (2022). The safety of propofol versus sevoflurane for general anesthesia in children: a meta-analysis of randomized controlled trials. Front Surg.

[12] Bock M, Haselmann L, Böttiger BW, Motsch J (2007). Priming with rocuronium accelerates neuromuscular block in children: a prospective randomized study. Can J Anaesth.

[13] Taivainen T, Meretoja OA, Erkola O, Rautoma P, Juvakoski M (1996). Rocuronium in infants, children and adults during balanced anaesthesia. Paediatr Anaesth.

[14] Kumar A, Kumar A, Bharti AK, Choudhary A, Hussain M, Dhiraj S (2023). A randomized double-blind comparative study of the intubating conditions and hemodynamic effects of rocuronium and succinylcholine in pediatric patients. Cureus.

[15] Janse van Rensburg E, Indiveri L, Mogane P (2025). The perioperative use of dexmedetomidine in paediatric patients. Children (Basel).

[16] Xiao A, Feng Y, Yu S, Xu C, Chen J, Wang T, Xiao W (2022). General anesthesia in children and long-term neurodevelopmental deficits: A systematic review. Front Mol Neurosci.

